# Criteria deemed important by ICU patients when designating a reference person

**DOI:** 10.1016/j.jointm.2022.04.005

**Published:** 2022-06-27

**Authors:** Jean-Pierre Quenot, Nicolas Meunier-Beillard, Eléa Ksiazek, Caroline Abdulmalak, Fiona Ecarnot, Jean-Baptiste Roudaut, Pascal Andreu, François Aptel, Marie Labruyère, Marine Jacquier, Jean-Philippe Rigaud

**Affiliations:** 1 Department of Intensive Care, University Hospital François Mitterrand, Dijon 21000, France; 2 Lipness Team, INSERM Research Centre LNC-UMR1231 and LabEx LipSTIC, University of Burgundy, Dijon 21000, France; 3 INSERM CIC 1432, Clinical Epidemiology, University of Burgundy, Dijon 21000, France; 4 Espace de Réflexion Éthique Bourgogne Franche-Comté (EREBFC), Dijon 21000, France; 5 DRCI, USMR, CHU Dijon Bourgogne, Dijon 21000, France; 6 Department of Intensive Care, Centre Hospitalier William Morey, Châlon sur Saône 71000, France; 7 Department of Cardiology, EA3920, University of Franche-Comté, University Hospital Besancon, Besancon 25000, France; 8 Department of Intensive Care, Centre Hospitalier de Dieppe, Dieppe 76202, France; 9 Espace de Réflexion Éthique de Normandie, University Hospital Caen, Caen 14000, France

**Keywords:** Reference person, Intensive care unit, Questionnaire

## Abstract

**Background:**

We investigated the criteria that hospitalized patients in intensive care units (ICUs) deem important when designating relatives who are best qualified to interact with the caregiving staff.

**Methods:**

We conducted an exploratory, observational, prospective, multicenter study between March 1, 2018, and October 31, 2018, within two ICUs. A 12-item questionnaire was distributed to patients in the ICUs by the investigating physicians. Patients were considered eligible if they had a good understanding of the French language and if they had not officially designated surrogates before ICU admission.

**Results:**

Seventy-one patients whose average age was 63.9± 17.3 years, of whom 21 (29.5%) were females, completed the questionnaire. The average Charlson comorbidity score was 2.5 ± 2.4, and the average Simplified Acute Physiology Score (SAPS II) was 39.8 ± 16.5. The main etiology was respiratory infection (40.8%), followed by sepsis (23.9%). The most important criteria identified by patients when selecting reference persons were a good knowledge of the patient's wishes and values, an emotional attachment to the patient, and being a family member.

**Conclusion:**

Our findings reveal that ICU patients considered the following criteria to be critical when designating reference persons: knowledge of their wishes and the existence of emotional and family attachments.

## Introduction

The majority of patients in intensive care units (ICUs) are unable to express themselves either because of the severity of their disease or because they are sedated. Communicating with patients, providing information about their health status, and obtaining consent for procedures or research is therefore challenging, if not impossible, for medical personnel during most of the patients’ stay in the ICU.[1], [2] The concept of a surrogate was introduced in 2002 within French legislation and relates to the rights of patients and the quality of healthcare delivered within the French healthcare system. Within this legislation, a surrogate is defined as a person, designated by the patient, who can accompany the patient throughout the process of healthcare and can also testify to the patient's wishes in case the latter becomes unable to express their wishes themselves.[3], [4], [5] A surrogate is designated for the entire duration of the hospital stay and should ideally be designated before admission. However, pre-admission designation rarely occurs, especially for ICU admissions, which often entail emergency situations arising from life-threatening health conditions.

Despite the dispositions of successive French laws regulating patients’ rights, this important aspect of the patient–caregiver relation remains problematic. Two previous French studies[6], [7] explored criteria that could be used to identify specific individuals among the patients’ relatives who were considered best suited to act as the intermediary in case no official surrogate had been designated before the patient's admission to the ICU. The defining criteria were identified using a questionnaire completed by caregivers^[7]^ and by the family members of ICU patients.^[6]^ The criteria identified by caregivers in the questionnaire items as being the most important for selecting a reference person were having a good knowledge of the patient's wishes and values, an emotional attachment to the patient, and an adequate understanding of the patient's clinical history.^[7]^ Family members selecting a surrogate from among themselves also identified the same criteria as determinants of their choices,^[6]^ although the criterion of being a family member was considered more important by the families than by the caregivers in the choice of a surrogate. A family centered approach^[8]^ emphasizes these criteria for selecting a surrogate, not least because the healthcare team will have to discuss various ethical questions with that person during the patient's ICU stay. Such questions may include but are not limited to end-of-life issues, the risk–benefit ratio of ICU care for the patient in terms of future quality of life[9], [10] the level of therapeutic engagement, and the development of a healthcare plan.

Before their admission to ICUs in France, patients rarely convey their wishes and desires regarding healthcare or end-of-life issues to their family members. The family members may therefore (consciously or unconsciously) make medical decisions for the patient without giving due consideration to all of the issues at stake or all of the possible consequences. In rare cases, family members may even put their own interests before those of the patient when making decisions on the patient's behalf.[11], [12] Furthermore, in the absence of a designated surrogate, it is often the spouse who gets designated “by default.” This unexpected role that falls to another family member can be a source of mental stress for them, as they are not prepared for the responsibility that it entails. Consequently, the responsibility may induce anxiety, depression, post-traumatic stress disorder, or post-intensive care syndrome.[13], [14]

Building on previously published studies conducted among caregivers and family members,[6], [7] we aimed to elicit criteria from patients, after their discharge from the ICU that they would have used to choose a reference person.

## Methods

We carried out an exploratory, observational, prospective, multicenter study between March 1, 2018, and October 31, 2018. A questionnaire comprising 12 questions was developed by two senior ICU physicians and a sociologist using a methodology described elsewhere.[7], [15], [16], [17] The empirical data used to formulate the questionnaire items were obtained from a preliminary qualitative study conducted among 15 healthcare professionals^[7]^ working in a single ICU (7 nurses, 5 nurses’ aides, and 3 ICU physicians). The questionnaire was validated for use in the present study by a panel of 15 ICU patients through semi-directed interviews conducted after their discharge from the ICU. In the present study, the importance of each criterion relating to the role of the reference person was ranked by the patients. Criteria were rated using a scale ranging from 0 (*not important at all*) to 10 (*extremely important*). An average score >7 indicated that an item was important. The study was registered with ClinicalTrials.gov under the identifier NCT03261258.

### The questionnaire

The questionnaire was distributed to hospitalized ICU patients by the investigating physicians in two ICUs: a mixed ICU in a non-academic general hospital and a medical ICU in a university teaching hospital. All questionnaires were anonymous. Patients were eligible to participate in the study if they had a good understanding of the French language and if they had not previously appointed surrogates before their admission to the ICU.

The institutional review board (Comité de Protection des Personnes Est I) approved the protocol, which was deemed to constitute routine clinical practice. Information about the study was provided orally to each patient by the main investigator at each center. The patients were also given a printed document containing detailed information about the study. After the patients had been given time to consider the information and ask any questions, they were enrolled in the study, and their participation was noted in their medical files. French legislation does not require the provision of written informed consent. Patients may choose to opt-out of a study by submitting a written objection. The absence of this written objection implies that they are not opposed to participation. In compliance with French privacy laws, the collection of nominative data was approved by the national authority for the protection of privacy and personal data (Commission Nationale Informatique & Libertés), which is the legal authority responsible for the protection of individuals’ privacy and personal data.

### Data sources

The following data were recorded for each patient: socio-demographic characteristics, co-morbidities evaluated using the Charlson Comorbidity Index,^[18]^ Katz's Activities of Daily Living,^[19]^ disease severity calculated using the Simplified Acute Physiology Score (SAPS) II,^[20]^ the Sequential Organ Failure Assessment (SOFA) score^[21]^ at the time of ICU admission, the length of the ICU and hospital stay, and death in the ICU or hospital.

Dedicated clinical research assistants collected all the data using a standardized electronic case reporting form. Automatic checks were generated for missing or incoherent data. Data used for clinical and epidemiological investigations were independently managed by the centers.

### Statistical analysis

The study population was described using continuous variables, expressed as means and standard deviations, and categorical variables, expressed as numbers and percentages. Responses to each question received from all the respondents were graphically represented using means and standard deviations. Relations between item responses were subsequently examined using Pearson's correlation coefficient, followed by principal component analysis (PCA) conducted on standardized data to summarize data from each item and define response profiles. The choice of the number of axes to be retained was based on the scree plot and “elbow” criterion. The interpretation of the PCA axis was based on variables that were well represented on the axis (cos2 > 30), with contributions ≥10% in the construction of the axis. We applied simple linear regression or one-way analysis of variance (ANOVA) models to assess the association between each PCA axis and different population characteristics (sex, age, SAPS II, SOFA score, and length of ICU stay).

A *P*-value of <0.05 was considered statistically significant. All analyses were performed using SAS version 9.4 (SAS Institute Inc., Cary, NC, USA).

## Results

**Figure 1:**
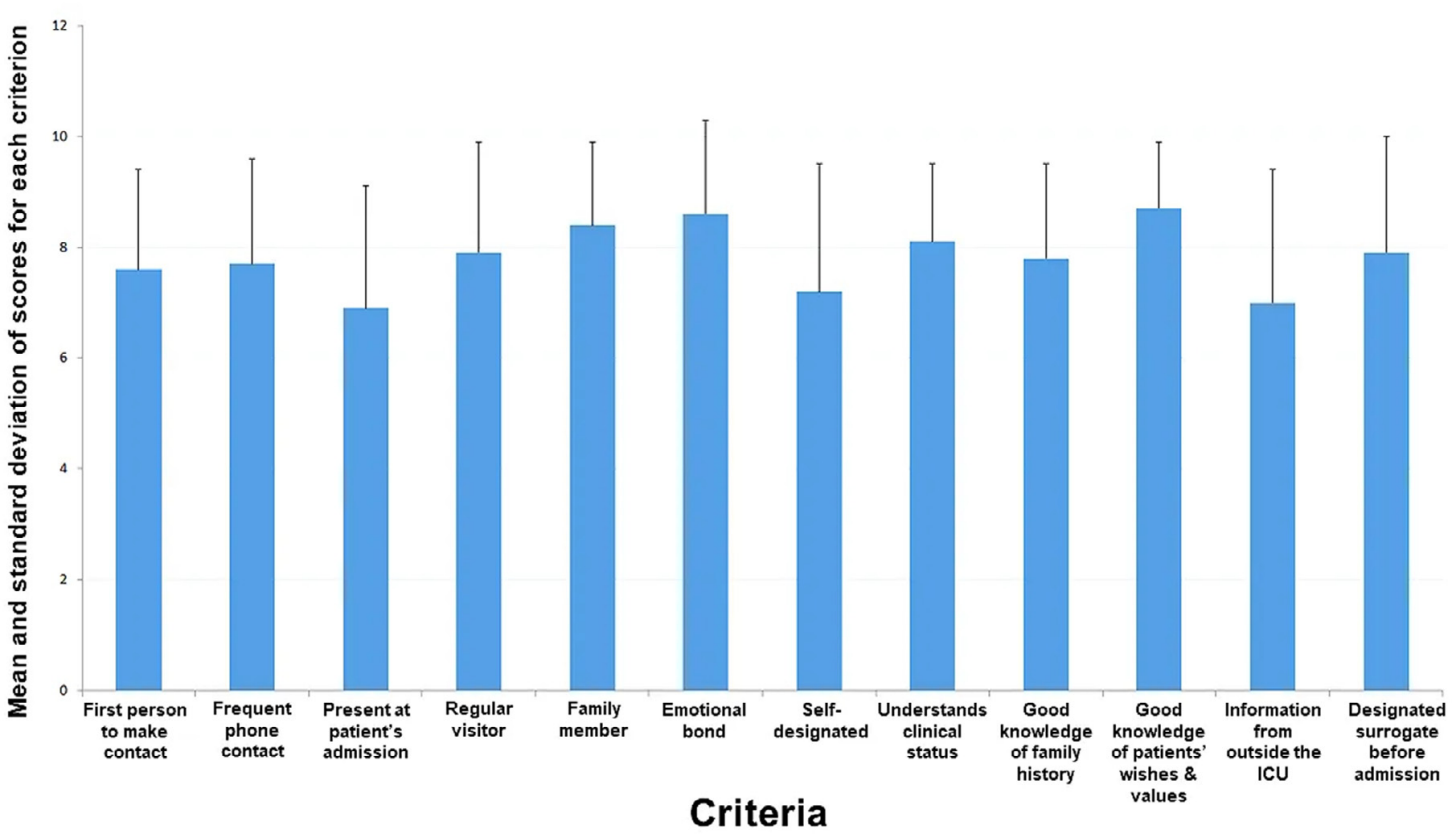
Average and standard deviations of scores for the importance assigned by patients to each questionnaire item (each criterion was rated on a scale from 0 to 10). ICU: Intensive care unit.

A total of 71 patients consecutively admitted to the two participating ICUs completed the questionnaire. The average age of the patients was 63.9 ± 17.3 years. The main characteristics of the 71 patients are presented in Table 1. The following items were identified by the respondents as being the most important criteria for selecting a reference person: being a family member, having an emotional attachment with the patient, and having a good knowledge of the patient's wishes and values [Figure 1]. Moderate correlations were identified among the following variables: (1) being the first person to make contact and to engage in frequent telephonic contact (*r*=0.57); (2) frequent telephonic contact and being a regular visitor (*r*=0.59); (3) being a regular visitor and information from the hospital admissions office about the existence of a surrogate (*r*=0.57); (4) being a family member and having an emotional bond with the patient (*r*=0.51); (5) having an emotional bond with the patient and a good knowledge of their family history (*r*=0.53); (6) having an emotional bond with the patient and obtaining information from the hospital admissions office (*r*=0.50); and (7) having information from the hospital admissions office and being the designated surrogate before admission (*r*=0.56). All other correlations were found to be weak.

**Table 1:** Characteristics of the 71 patients who completed the questionnaire.

Variables	Data (*n*=71)
Age (years)	63.9 ± 17.3
Sex: female	21 (29.5)
Charlson comorbidity score	2.5 ± 2.4
SAPS II score	39.8 ± 16.5
SOFA score	5.5 ± 3.8
Katz's activities of daily living	5.0 ± 1.4
Main reason for ICU admission	
Respiratory issue	29 (40.8)
Sepsis	17 (23.9)
Cardiac issue	14 (19.7)
Renal issue	5 (7.2)
Neurological issue	4 (5.6)
Other reason	2 (2.8)
Length of ICU stay (days)	9.2 ± 17.8
In-ICU death	2 (2.8)
In-hospital death	2 (2.8)

Data are expressed as *n* (%) or means ± standard deviations.

ICU: Intensive care unit; SAPS II: Simplified Acute Physiology Score II; SOFA: Sequential Organ Failure Assessment.

The first three PCA axes summarized 57.9% of the overall variability and were retained. The first group of criteria (Axis 1) comprised frequent telephonic contact, being a regular visitor, being the designated surrogate before admission, and having an emotional bond with the patient. Patients with high scores on this axis viewed frequent contact (physical or telephonic), being the designated surrogate before admission, and having an emotional bond as important criteria in the choice of a reference person. This axis explained 35.2% of the overall variability. The second group of criteria (Axis 2), which accounted for 11.5% of the overall variability, consisted of having a sound understanding of the patient's clinical situation and a good knowledge of the patient's wishes and values. Patients with high scores on this axis considered these two criteria to be the most important ones in determining their choices of reference person.

The third group (Axis 3), which summarized 11.2% of the overall variability, comprised a single criterion, namely being the first person to make contact with the ICU staff to get an update on the patient's state of health. Patients with high scores on this axis considered this criterion to be important for choosing a reference person. Table 2 depicts the quality of representation and the contributions of each item to each of these three axes. No significant associations were found between the PCA axes and patients’ characteristics.

**Table 2:** Contribution of the individual questionnaire items to each of the three axes retained following principal components analysis.

Item	Axis 1		Axis 2		Axis 3	
	Cos2	Contribution	Cos2	Contribution	Cos2	Contribution
First person to make contact with the ICU to ask about patient	0.28	6.55	0.06	4.49	0.31*	22.92*
Frequent telephonic contact	0.56*	13.35*	<0.01	0.12	0.14	10.48
Present at patient's admission	0.35*	8.38*	<0.01	0.21	0.15	11.48
Regular visitor	0.55*	12.98*	<0.01	0.03	<0.01	<0.01
Family member	0.42	9.88	0.05	3.66	<0.01	0.03
Emotional bond	0.51	12.09	<0.01	<0.01	0.12	8.77
Self-designated	0.21	4.93	0.05	3.94	0.11	8.23
Understands clinical status	0.02	0.51	0.49*	35.44*	0.07	4.97
Good knowledge of family history	0.28	6.64	0.26	19.13	0.22	16.45
Good knowledge of patient's wishes and values	0.04	0.94	0.35*	25.69*	0.16	11.52
Information from admissions office re surrogate status	0.54*	12.71*	0.01	0.72	0.03	2.32
Designated surrogate before admission	0.47*	11.05*	0.09	6.58	0.04	2.83

⁎ Values for variables with good representation on the axis (cos2 > 30) and with a contribution ≥10% in the axis construction.

## Discussion

We identified the following criteria considered important by hospitalized patients in ICUs when designating a reference person: being a family member, having an emotional attachment with the patient, and having a good knowledge of the patient's wishes and values. Two of these criteria, namely having an emotional attachment with the patient and having a good knowledge of the patient's wishes and values have previously been identified by healthcare professionals as well as by the families of ICU patients.[6], [7] We can therefore hypothesize that “being a family member,” which was identified as an important criterion by the patients, largely coincides with “knowing the patient's clinical history,” which was identified as important by the healthcare professionals and by the patients’ families.[6], [7] Indeed, it is because they meet this latter criterion that family members are usually involved in discussions with the physician and healthcare team. When a patient is incapacitated and unable to express themselves, their family members are generally considered to be the preferred reference persons for decision-making, especially in situations in which decisions must be made about the level of therapeutic engagement and/or the possible pursuit or discontinuation of intensive care.[10], [22], [23]

In light of our findings and those of previous studies on healthcare professionals and the families of ICU patients,[6], [7] it is reassuring for the caregiving team to know that throughout the patient's stay, medical information will be shared with reference persons whose profiles meet the requirements of everyone within the healthcare triad, namely the patient, the family, and the healthcare team. Even when a surrogate had not previously been designated before the patient's admission to the ICU, these findings indicate that the healthcare team, the family, and the patient are all in agreement that the selection of the reference person should be based on this individual's solid knowledge of the patient and a concern to ensure that the patient gets the treatment they would have wanted.^[24]^ This knowledge includes that of the patient's life history (usually best known to family members and/or close friends or relatives), as such information can help to guide the decision on whether or not to proceed with intensive care and subsequent treatment choices, such as initiating life support therapies (or not).^[25]^ It should be noted that while French law allows patients to designate surrogates, it is not mandatory for them to do so. Similarly, there are dispositions that allow for the preparation of advance directives, but few patients actually engage in these preparations or even express their end-of-life wishes to family members.^[4]^ Accordingly, in the specific context of ICU admission, as long as the reference person meets certain criteria, and even if that person has not officially been designated as a surrogate, they can act as the intermediary on behalf of the patient, receive medical information about the patient from the healthcare team, and participate in treatment decisions for the patient without difficulty. Therefore, in the absence of an officially designated surrogate and/or advance directives, the patient's medical information can be shared with their relatives without any major restrictions and without blatant disregard for the patient's wishes.

Given that patients, relatives, and healthcare providers all have similar views regarding the qualities of a suitable surrogate, it may be time to consider encouraging open and transparent discussion among patients, families, and healthcare providers regarding the pertinence of ICU admission, appropriate levels of therapeutic engagement, and the duration of that engagement. Ideally, such discussions should take place before the occurrence of an acute event. Accordingly, ICU physicians should be called upon to provide their insights when a patient's care goals are being discussed,^[22]^ especially when progressive deterioration of the patient's health status is likely to warrant intensive care.[26], [27], [28], [29], [30], [31]

The findings of this study open interesting avenues for future research. The first is to rank the importance of different criteria using, for example, a concept mapping methodology during a follow-up session. The second is to conduct a larger scale, multicenter study to validate the criteria identified here and to ensure that the findings are not center-specific.

## Limitations

This study had several limitations. First, it was performed in only two ICUs with relatively small numbers of (predominantly male) respondents. Moreover, it was limited to French ICU patients, a majority of whom had no officially designated surrogates. In-hospital survival rates were also very high within our study sample. Nonetheless, the narrow confidence intervals around the estimates for each of the items support the view that a larger number of respondents would not significantly alter the results. Second, other patient characteristics, such as their life trajectories, socioeconomic status, and the existence of advance directives, which were not recorded here, may have impacted the results. Third, we cannot exclude the possibility that our questionnaire was not exhaustive and that other factors exist that were not taken into account. Finally, the stress generated by the ICU environments in which the patients completed the questionnaires may have influenced their responses. Furthermore, although responses from patients and relatives were not collected simultaneously, we cannot exclude the possibility that there may have been some contamination bias. Conversely, our study also had several strengths. The questionnaire was developed using methodologically robust qualitative methods based on semi-directive interviews conducted until saturation was reached. In addition, the data recorded here can be deemed robust, as they explained >60% of the total variance across three PCA axes, with fairly strong correlations (*r* >0.5).

## Conclusions

The findings of this study revealed that the criteria considered by patients to be the most important ones for designating reference persons for ICU patients are knowledge of the patient's wishes and the existence of emotional and family attachments to the patient. They endorse the findings of previous studies conducted on the same topic among healthcare providers and patients’ relatives.

## Funding

This research did not receive any specific grant from funding agencies in the public, commercial, or not-for-profit sectors.

## Conflicts of Interest

The authors declare that they have no known competing financial interests or personal relationships that could have appeared to influence the work reported in this paper.
